# Evaluating experimentally the viability of employing hybrid nanofluids as an operating fluid in a shell-and-tube heat exchanger

**DOI:** 10.1038/s41598-025-87149-7

**Published:** 2025-02-03

**Authors:** Amr M. Hassaan

**Affiliations:** Mechanical Engineering Department, Higher Technological Institute (HTI), Tenth of Ramadan City, Egypt

**Keywords:** Hybrid nanofluids, Convection, Shell and tube, Experimental study, Heat transfer, Heat exchangers, Mechanical engineering, Nanoscale materials, Nanoparticles

## Abstract

The use of hybrid nanofluids (HNF) in heat transfer applications has become the subject of studies during recent periods. Its ability to transfer heat is superior to single nanofluids, and this has been proven in many research. Shell and tube heat exchanger is one of the most common types and are therefore always under research to improve their performance. This study is a practical study that examines the effectiveness of HNF in their use as operating fluids in shell-and-tube heat exchangers instead of traditional operating fluids. A laboratory heat exchanger was used to complete this study. A group of HNF (MWCNTs- Al_2_O_3_/water) was synthesized at different concentration rates ranging from 0.4 to 2%. Tests were conducted for Reynolds values in the range from 2500 to 12,560. By measuring the variables in the tests and calculating some factors the results showed that, in comparison between the use of HNF and distilled water, it was found that there is an increase in the value of the Nusselt number for the HNF over the distilled water in a range of 10- 28.5%. The effectiveness increased when using the HNF by up to 22.7% compared to distilled water. Through experiments, an equation was deduced to calculate the value of the Nusselt number. There is good agreement between the results of this research and previous literature.

## Introduction

Regardless of its type and shape, one of the most crucial mechanical devices in use across several sectors is the heat exchanger. It is an essential tool because of its many benefits in the heating and cooling processes. Heat exchangers (HEs) are responsible for providing enhanced thermal efficiency. In several branches of contemporary industry, HEs are now indispensable parts^1^. The shell-and-tube heat exchanger (STHX) is among the many HEs used in industries. Several tubes inside a large, high-pressure cylindrical tank make up this HE^2,3^. Numerous attempts have been made to enhance the thermo-hydraulic efficiency of these HEs because of their widespread use in industry^4^. In the context of contributing to developing the effectiveness of the STHX, nanofluids were used instead of traditional fluids^5^. These fluids are characterized by their ability to transfer higher heat rates than ordinary fluids due to their improved physical properties^6–9^. To enhance the heat transfer capabilities of standard heat transfer fluids like mineral oil, ethylene glycol, and water, one or more types of nanosized particles are introduced and disseminated. In keeping with this idea, it is worth noting the following overview of some earlier research on the improvement of heat transfer from different studies utilizing nanofluids:

Different water-based HNF were used as coolant in Sumit et al.‘s^10^ evaluation of the shell and tube condenser systems. Results demonstrated that employing Al_2_O_3_ + MWCNT hybrid nanofluid may lower operating costs by 11.08% and the coolant mass flow rate by up to 3.6%, respectively. A numerical simulation of STHX, MWCNT-water nanofluid, using CFD was conducted by Hu et al.^11^. Increased concentration of the nanofluid led to a greater improvement in heat transfer performance because MWCNT-water nanofluids had superior thermal conductivity than base fluid. Hassaan^12^ studied the use of a HNF (Al_2_O_3_ + MWCNT/water) in a plate heat exchanger (PHE). According to the findings, the overall heat coefficient could improve by 6–97%, depending on the concentration of nanoparticles in comparison to distilled water. At a ratio of 0.5, the heat transfer coefficient improved by 15.2%, according to research by Atul et al.^13,14^ on the effect of Al_2_O_3_-MWCNT nanoparticle volume ratios on the thermodynamic performance of PHEs. Using Al_2_O_3_/water and MWCNT/water nanofluids as thermal fluids, Huang et al.^15^ examined the thermal behavior of a PHE with chevron-type microchannels. The outcomes showed that HTC for both types of nanofluids had only slightly improved. In a shell and tube heat exchanger, Anitha et al.^16^ employed Cu/water and Al_2_O_3_-Cu/water nanofluids. At higher Reynolds numbers, they saw a 139% increase in the heat transfer coefficient for HNF and a 25% increase for Cu nanofluids. Through numerical investigation, Zakir et al.^17^ determined that while natural convection is decreased at high volume concentrations of SiO_2_ nanoparticles, the charging rate increases by 41% at 5% volume concentrations of the particles. Using several water-based HNF as coolants, Sumit et al.^10^ evaluated the shell and tube condenser’s performance. According to the results, utilizing an Al_2_O_3_ + MWCNT HNF can lower the coolant mass flow rate and operating costs by up to 3.6% and 11.08%, respectively. According to Mohammad et al.^18^, for both laminar and turbulent flow, the Fe_2_O_3_ + CNT HNF has a higher heat transfer coefficient than the base fluid. Utilizing varying concentrations of TiO_2_-CNT/water HNF under conditions of laminar flow, Megatif et al.^19^ examined the heat transfer coefficients and thermal performance for STHX. Using varied concentrations of Fe_2_O_3_-CNT/water HNF, Aghabozorg et al.^20^ investigated the heat transfer characteristics for horizontal STHX under numerous flow conditions. The results showed improvements in the heat transfer coefficients for laminar and turbulent flow conditions. Al_2_O_3_-Ag/water HNF performed better than other HNF under study, according to Singh and Sarkar’s^21^ investigation of the thermodynamic performance of STHX utilizing a variety of HNF. The thermodynamic performance of STHX in turbulent and laminar flow conditions was studied by Esfahani and Languri^22^ employing concentrations of graphene oxide HNF. ZnO/water has the most effectiveness whereas SiO_2_/water HNF have the lowest, according to Shahrul et al.‘s^23^ comparison of the performance of STHX utilizing oxide HNF such as CuO/water, Al_2_O_3_/water, TiO_2_/water, Fe_3_O_4_/water, and SiO_2_/water. The effect of baffle spacing on the efficiency of STHX was investigated statistically by Zebua and Ambarita^24^.

### The current study’s contribution

It is clear from previous literature in general that the performance of hybrid nanofluids in heat transfer processes has a very positive impact on all heat transfer systems. However, the scientific passion of researchers in using various nanofluids, whether single or hybrid, remains an important research path for researchers in the field to know the extent of the impact of each type individually.

In this research, the researcher decided to use a nano-hybrid fluid (MWCNTs- Al_2_O_3_/water) with a mixing ratio of )50:50( to determine its performance in the heat transfer process in a shell-and-tube heat exchanger with different volumetric concentrations and also with a difference in the temperature of the fluid and the speed of fluid flow inside the pipes. The materials were chosen because, through research in the literature and to the best of the author’s knowledge, there is not a sufficient amount of use of this hybrid fluid in the same way as the study.

## The experimental rig for research

### Test rig description

**Fig. 1:**
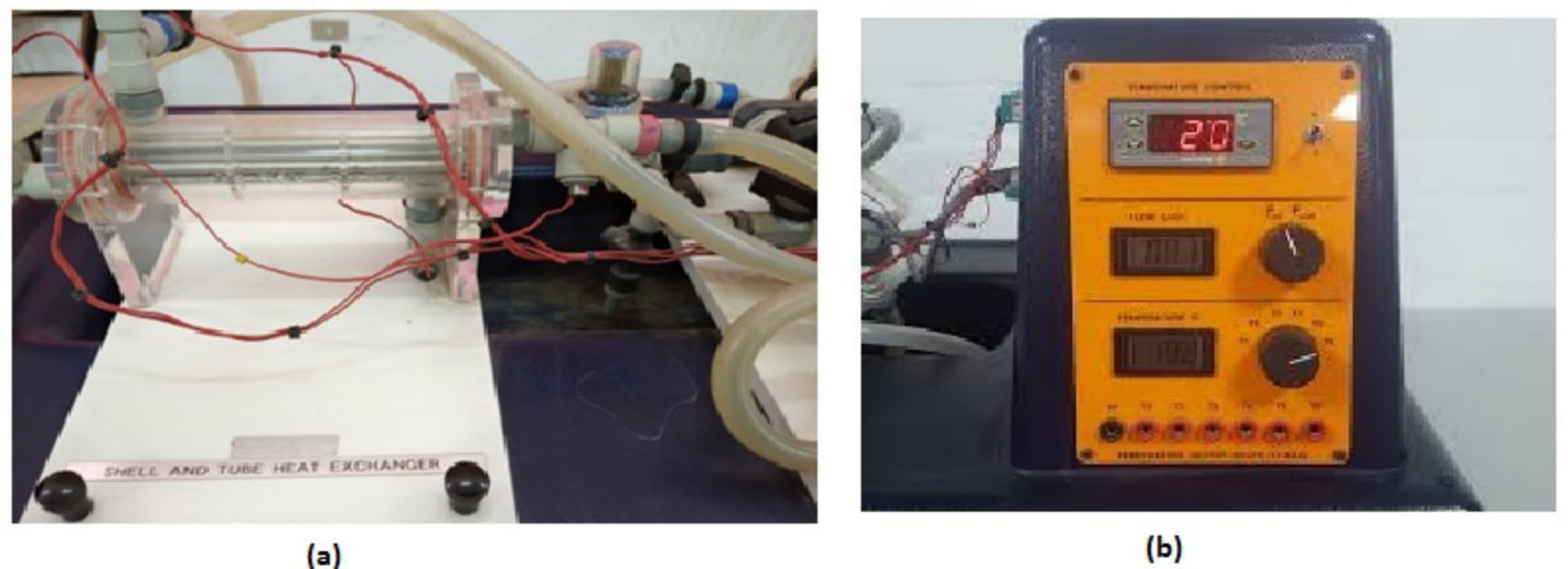
(**a**) Image of SHTX used in the study (**b**) service unit for the heat exchanger.

Figure 1(a) shows a picture of the heat exchanger used in this study. The exchanger is a laboratory heat exchanger produced by Armfield, model HT33. The heat exchanger must be connected to a service unit Fig. 1(b) created by the same company and carrying the HT30X model. The unit used supplies the heat exchanger with hot and cold fluid, and through it, the temperature of the hot fluid can be controlled. The unit also contributes to the possibility of controlling the fluid flow rate, and through it, the temperatures of the hot and cold fluid entering and exiting the heat exchanger can be known. The tube-shell heat exchanger used in this study contains seven horizontally parallel tubes with the same geometric dimensions and are made of stainless steel. The dimensions of a single tube are as follows: the inner diameter of the tube is 5.15 mm, the outer diameter of the tube is 6.35 mm, and the length of the tube is 144 mm. The hot fluid, whether HNF with different concentrations or pure distilled water, flows inside the stainless-steel tubes of the exchanger. The outer shell of the heat exchanger, which contains the tubes, is made of acrylic and has two transverse baffles that are also made of the same shell material, to reduce heat loss. The purpose of the two baffles is to increase the speed and spread of the cooling water, which contributes to increasing the rate of heat transfer inside the exchanger. All connections and covers of the exchanger are made of plastic to reduce thermal loss during operation. Four K-type thermocouple cables were placed at the hot and cold fluid inlets and outlets to measure the temperatures of the fluids circulating in the exchanger. A simple modification was made to the connections for the working fluid (hot fluid) used in the exchanger, with the aim of providing the device with a manometer connected to the inlet and outlet of the hot fluid to determine the loss in pressure during flow inside the exchanger.

The service unit for the HT30X heat exchanger is the basic working unit as the company produced it to operate its heat exchangers. The unit contains the power unit which is a tank with an electric heater to heat the hot fluid that will pass through the exchanger. There is a header outside the unit to know the water level inside the tank. The power unit also contains a gear pump to circulate the hot working fluid through the heat exchanger. The temperature of the hot fluid is set to the required temperature for the experiment by a thermostat located in the service unit. The cold water that passes through the exchanger is the tap water in the laboratory while it is drained directly into the laboratory drain. The service unit contains a turbine flowmeter to measure the flow rate of both fluids directly. There is also a valve in the unit on each path for the hot and cold fluids to control the flow rate. The unit is also equipped with a temperature gauge and a selector to connect the thermocouples to it and identify the temperature of the fluids through it.

### Production of hybrid nanofluids

**Fig. 2:**
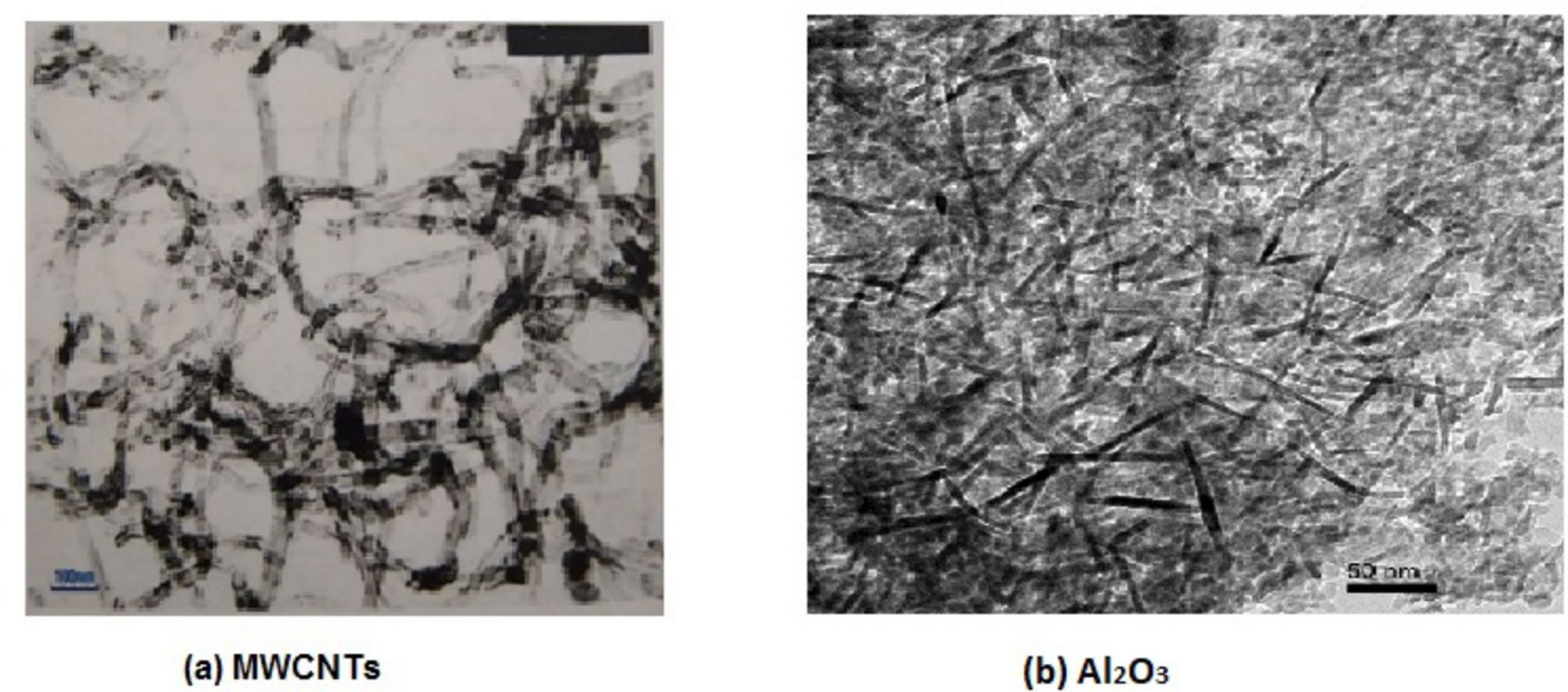
A TEM image for the used nanoparticles.

HNF is a conventional fluid to which nanoparticles of two or more materials are added to improve the fluid properties. The present research used Al_2_O_3_ particles with MWCNTs particles in a ratio of 50:50 and mixed with distilled water but at different volume concentrations. This ratio was chosen based on what was mentioned in some previous literature. MWCNTs and Al_2_O_3_ nanoparticles are seen in a TEM picture in Fig. 2. Al_2_O_3_ was purchased from Sigma-Aldrich Chemicals in the USA, while MWCNTs were obtained from Cheap Tubes Company in the USA. The properties of the particles used according to the supplier are listed in Table 1. The HNF was fabricated with five different volumetric concentrations (0.4%, 0.8%, 1.2%, 1.6%, 2%) but at a fixed mixing ratio.

**Table 1:** Particles specifications.

MWCNTs used specifications		AL_2_O_3_ used specifications	
Purity	> 90%	Particle size	≤ 50 nm
Length	10–30 μm	Specific heat	880 J/Kg K
Outer diameter	20–40 nm	Density	3690 kg/m^3^
Specific heat	790 J/Kg K		
Density	2100 kg/m^3^		

**Fig. 3:**
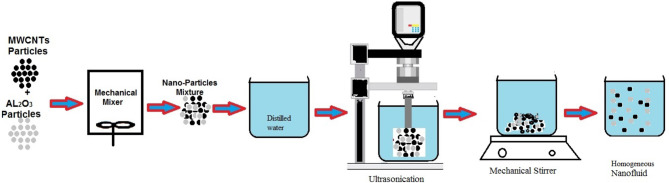
Diagrammatic representation of the preparation of nanofluids.

Figure 3 illustrates the steps involved in manufacturing the fluids under study. Based on the targeted concentration Using Eq. (1)^25^, the weight of the nanoparticles needed for each volume concentration was determined. The amount of distilled water with which the particles will be mixed is also considered. It was a fixed amount for all concentrations used and was estimated at 2.5 l. The particles are mixed for five minutes in a mechanical mixer before being placed in the base fluid. One of the drawbacks of using nanofluids is the aggregation or sedimentation of particles after mixing. This problem can be overcome by exposing the mixture to ultrasonication for 40 min. After that, the entire mixture is placed on a mechanical stirrer for 8 h to complete mixing. Before placing the created fluid in the research device, the properties of the new fluid must be known and calculated using the equations listed below.

1 $$\:\text{V}\text{o}\text{l}\text{u}\text{m}\text{e}\:\text{c}\text{o}\text{n}\text{c}\text{e}\text{n}\text{t}\text{r}\text{a}\text{t}\text{i}\text{o}\text{n},\:\hspace{0.17em}\times\:\hspace{0.17em}100\text{\%}\:=\:\left[\frac{\left(\frac{{W}_{p}}{{\rho\:}_{p}}\right)}{\left(\frac{{W}_{p}}{{\rho\:}_{p}}\right)+\left(\frac{{W}_{w}}{{\rho\:}_{w}}\right)}\right]$$

where ρ_p_ is the nanoparticle density, ρ_w_ is the water density, and Φ is the volume concentration %.

The Pak and Cho formula^26^ is used to determine the density of nanofluid in the following manner:

2 $${\rho _{{\text{nf}}}}={\text{ }}({\text{1}} - \Phi ){\rho _{\text{w}}}\,+\,\Phi \,{\rho _{\text{p}}},$$

The specific heat capacity of nanofluid may be computed using the following formula^26^.

3 $$\:\text{C}\text{n}\text{f}\:=\:\left(1-\frac{\varPhi\:}{100}\right){C}_{w}+\:\frac{\varPhi\:}{100}\:\:{C}_{p}$$

The following formula^27^ can be used to determine the absolute viscosity:

4 $$\:{\upmu\:}\text{n}\text{f}\:=\:\left(1+\frac{5\varPhi\:}{2}\right){\mu\:}_{w}$$

Using the Maxwell model, the following formula^28^ may be used to determine the heat conductivity:

5 $$\:\text{K}\text{n}\text{f}\:=\:\frac{{k}_{p}+{2k}_{w}+\:\varPhi\:({k}_{p}-{k}_{w})}{{k}_{p}+{2k}_{w}-\:\varPhi\:\left({k}_{p}-{k}_{w}\right)\:\:}\:\text{k}\text{w}\:$$

### Experimental procedures

**Fig. 4:**
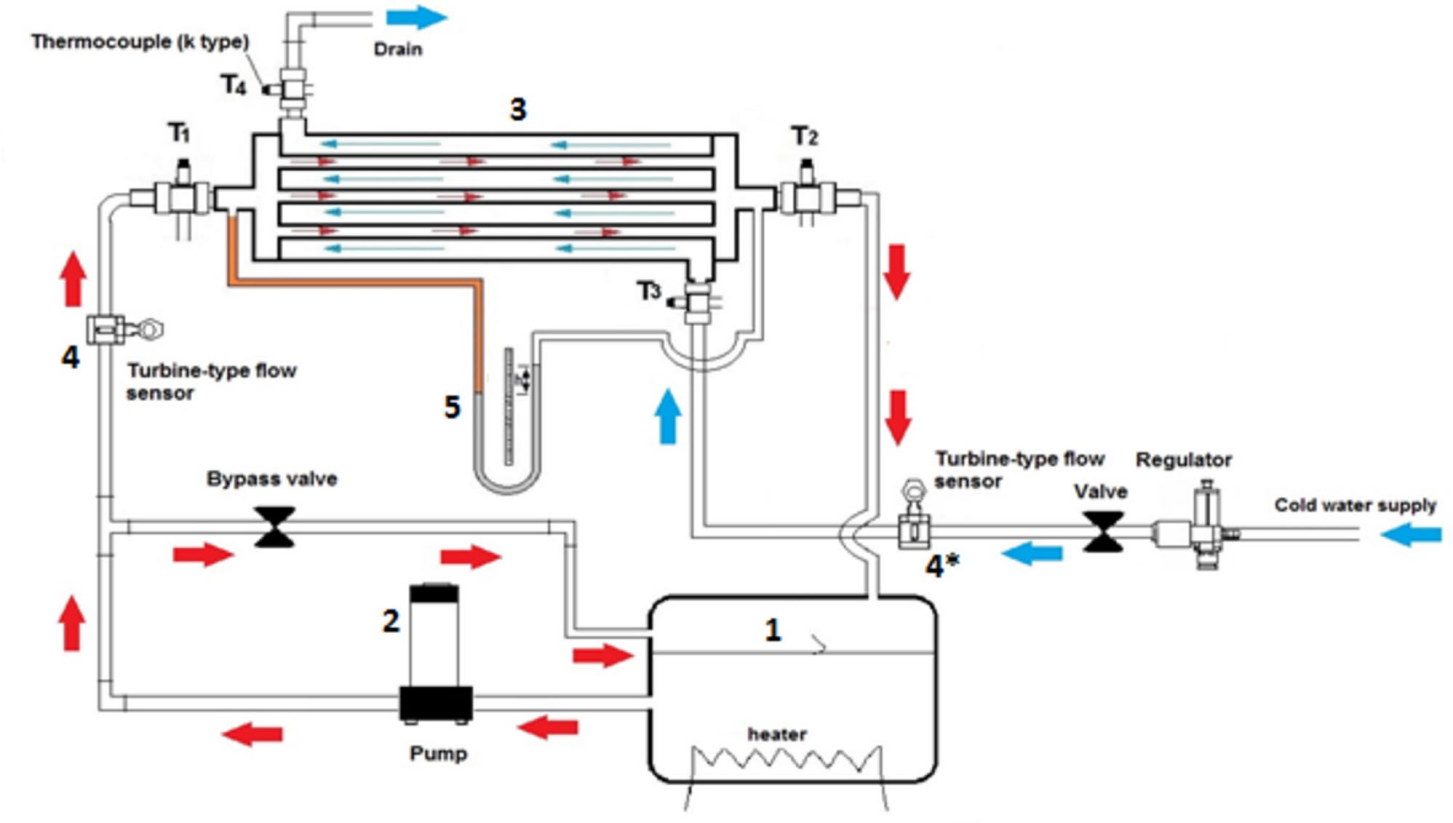
Figure schematic of the experimental test apparatus.

Figure 4 shows a schematic drawing of the research device used in the experiments with its components. These are the steps followed to prepare the experiment.

- After preparing the test fluid, we fill the tank (1) in the unit and turn on the heater.
- The thermostats are set to the required operating temperature.
- After reaching the operating temperature, the pump (2) is turned on to pass the fluid to the exchanger (3) through the connections in the unit.
- The flow rates for both fluids (hot and cold) are adjusted by the valves on each path.
- The device is left until the experiment reaches a steady state.
- After reaching a steady state, both flow rates are recorded by the turbine flow meter (4&4*) on each path, the inlet and outlet fluid temperatures, and the height of the manometer (5).
- The experiment data is recorded and processed as required and then used in the performance analysis process.

## Data reduction

Through the experimental data collected during the experiment, a set of factors were calculated in order to evaluate the thermal behavior of the HNF and the conventional fluid. The first of these factors was the RHT from or to the fluid, which was calculated as follows^29^:

6 $$\:\text{Q}\text{h}\text{f}\:=\:{m}_{hf}^{.}\:{C}_{p,\:\:hf\:}({T}_{hf,\:i}-{T}_{hf,o})$$

7 $$\:\text{Q}\text{c}\text{f}\:=\:{m}_{cf}^{.}\:{C}_{p,\:\:cf\:}({T}_{cf,\:o}-{T}_{cf,i})$$

where Q_hf_ represents the RHT for hot fluid (HNF or distilled water) & Q_cf._ is RHT for Cold fluid.

*m* is the mass flow rate of the fluid, C_p_ is specific heat of fluid (J/kg K), and $$\:{T}_{hf,\:i}$$, $$\:{T}_{cf,\:i,\:}$$$$\:{T}_{cf,o}\:$$and $$\:{T}_{h,f\:o}\:$$are the inlet and outlet temperatures of the hot and cold fluids.

8 $$\:\text{Q}\text{a}\text{v}\text{g}\:=\:\frac{{Q}_{hf}+{Q}_{cf}}{2}$$

Equation (9)^2^, which calculates the OHTC (U_h_), uses the average heat flow (Q_avg_) value of Q_hf_ and Q_cf._.

9 $$\:\text{U}\text{h}\:=\:\frac{\text{Q}\text{a}\text{v}\text{g}}{{\varvec{A}}_{\varvec{i}}\:\left(\frac{{\varvec{\varDelta\:}\varvec{T}}_{1}-{\varvec{\varDelta\:}\varvec{T}}_{2}}{\varvec{l}\varvec{n}\left(\frac{{\varvec{\varDelta\:}\varvec{T}}_{1}}{{\varvec{\varDelta\:}\varvec{T}}_{2}}\right)\:}\right)}$$

$$\:{\varDelta\:T}_{1}=\:{T}_{hf,\:i}-{T}_{cf,o;\:\:}\:{\varDelta\:T}_{2}=\:{T}_{hf,o}-{T}_{cf,i\:}$$

A STHX’s OHTC (U_h_), could be estimated as follow^2^:

10 $$\:\frac{1}{{{U}_{i}A}_{i}\:}=\:\frac{1}{{{h}_{o}A}_{o}\:}+\frac{\text{l}\text{n}\left(\frac{{D}_{o}}{{D}_{i}}\right)}{2\pi\:kL\:}+\:\frac{1}{{{h}_{i}A}_{i}\:}$$

where h is the HTC for inner and annulus-side (W/m^2^K), A is area (m^2^), and the inner tube material’s thermal conductivity, expressed as W/m.K, is denoted by k.

The HTC of the annulus-side (h_o_) can be computed using the formula (11):

11 $$\:\text{h}\text{o}\:=\:\frac{\text{Q}\text{a}\text{v}\text{g}}{A{(T}_{wall}-{T}_{cf,\:i})\:}$$

where T_wall_ = $\:\frac{{T}_{hf,\:i}-{T}_{hf,o\:}}{2\:};\:$D_h_ =D_o_ -D_i_ and A = πD_h_L.

The tubes-side heat transfer coefficient (h_i_) values from Eq. (10), the thermal conductivity of hf at mean temperature, and the inner tube diameter are substituted to obtain the non-dimensional Nusselt number (Nu_hf_) from Eq. (12)^25^.

12 $$\:\text{N}\text{u}\text{h}\text{f}\:=\:\frac{{\varvec{h}}_{\varvec{i}}\varvec{*}\:{D}_{i}}{{\varvec{k}}_{\varvec{h}\varvec{f}}}$$

The tube-side fluid nondimensional Reynolds number of hot fluid is determined using Eq. (13).

13 $$\:\text{R}\text{e}\text{h}\text{f}\:=\:{\left(\frac{\varvec{\rho\:}\varvec{*}\varvec{v}\varvec{*}\:{D}_{i}}{\varvec{\mu\:}}\right)}_{\text{h}\text{f}}$$

As seen below, the friction factor (ff) is computed using the mercury column’s pressure differential:

14 $$\:\text{f}\text{f}\:=\:\frac{{\Delta\:}\text{P}}{\left(\frac{{\varvec{L}}_{\varvec{i}}}{{\varvec{D}}_{\varvec{i}}}\right)\left(\frac{\varvec{\rho\:}{\varvec{v}}^{2}}{2}\right)}$$

where $\:\varvec{v}$ is the velocity of the hf (m/s)

Use of the following formula might yield STHX effectiveness^25^:

15 $$\:{\upepsilon\:}\:=\:\frac{\text{Q}\text{a}\text{v}\text{g}}{\varvec{m}\varvec{i}\varvec{n}\:[{m}_{hf}^{.}\:{C}_{p,\:\:hf\:\:}\:\:,\:{m}_{cf}^{.}\:{C}_{p,cf\:\:}]\left({\varvec{T}}_{\varvec{h}\varvec{f},\varvec{i}}-\:{\varvec{T}}_{\varvec{c}\varvec{f},\varvec{i}}\right)}$$

### Analysis of uncertainties

Since several parameters of varying precision were measured in this study, it is vital to examine how these parameters’ measurement uncertainty affected the findings. The impact of each parameter x_i_’s measurement uncertainty on the quantity R is computed if the quantity R is a function of the parameters x_1_, x_2_,……, xn as follows:

16 $$\:{\:\:\:\:\:\text{U}}_{\text{R}\text{i}\:}=\:\frac{{x}_{i}}{R}\frac{\partial\:R}{\partial\:{x}_{i}}\:{u}_{xi}$$

Since Ux_i_ represents measurement uncertainty, U_Ri_ is the greatest amount of uncertainty that can occur while computing a quantity, R is the quantity determined from measurable parameters, and x_i_ is the measurable parameter. The following formulas^30^ are used to determine the overall uncertainty of the quantity R caused by the parameter x_i_ errors. Table 2 shows the accuracy of the measurement instruments used in this research and the relative uncertainty of the research results.

17 $$R\,=\,f({x_1},{\text{ }}{x_{\text{2}}}, \ldots ,{\text{ }}{x_n}),$$

18 $$\:{\:\:\:\:\:\text{U}}_{\text{R}\text{i}\:}=\pm\:\left\{{\left(\frac{{x}_{1}}{R}\frac{\partial\:R}{\partial\:{x}_{1}}\:{u}_{x1}\right)}^{2}+{\left(\frac{{x}_{2}}{R}\frac{\partial\:R}{\partial\:{x}_{2}}\:{u}_{x2}\right)}^{2}+\dots\:+{\left(\frac{{x}_{n}}{R}\frac{\partial\:R}{\partial\:{x}_{n}}\:{u}_{xn}\right)}^{2}\right\}$$

19 $$\:{\:\:\:\:\:\text{U}}_{\text{R}}={\pm\:\left\{{\left({\:\text{U}}_{\text{R}1}\right)}^{2}+{\left({\:\text{U}}_{\text{R}2}\right)}^{2}+\dots\:+{\left({\:\text{U}}_{\text{R}\text{n}}\right)}^{2}\right\}\:}^{0.5}$$

**Table 2:** Uncertainties in experimental results.

Measured parameters		Derived relative uncertainty ranges through experimentation	
Parameter	Accuracy	Parameter	Parameter
Nanoparticles weight Volume flow rate Temperature U-tube manometer	*±* 0.001 g *±* 0.01 l/min *±* 0.1^o^C *±* 1 mm	Q h_i_ U_i_ Re Nu ffε	*±* 1.52% *±* 1.32% *±* 1.5% *±* 1.6% *±*2.1% *±* 4.2% *±* 2.7%

## Results and discussions

**Fig. 5:**
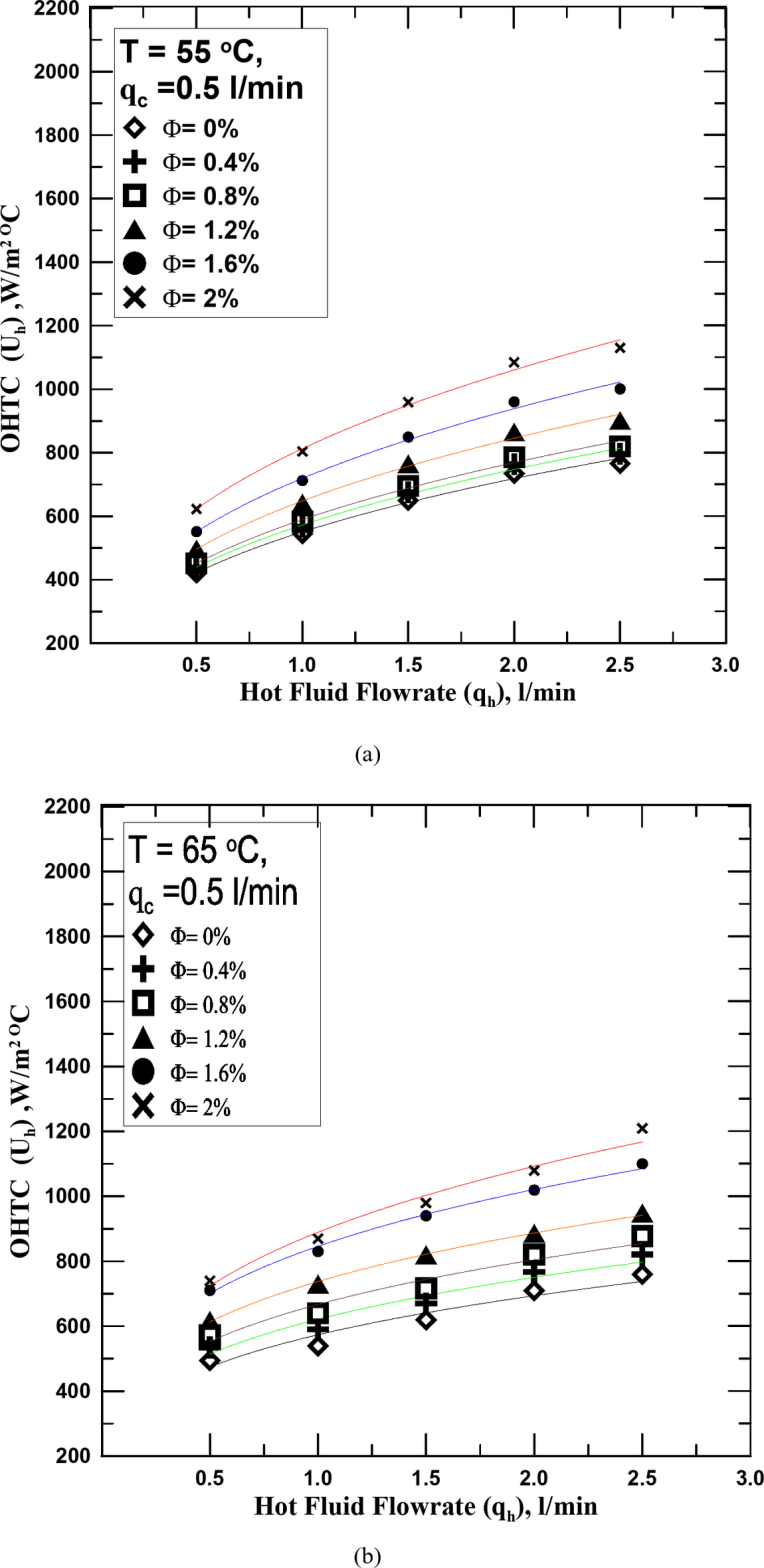

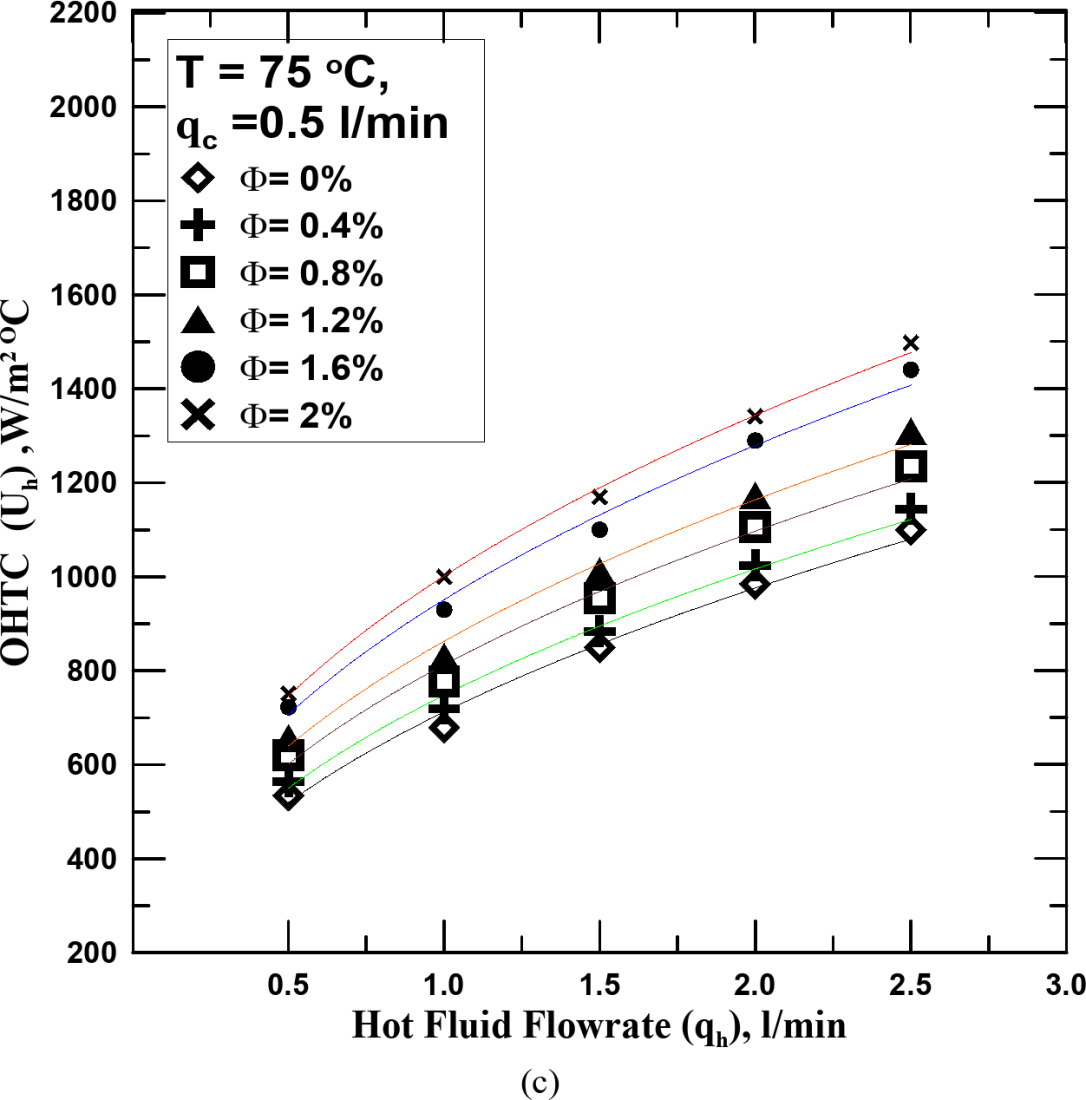
(**a**) U_h_ vs. q_h_ for diff. values of Φ at T = 55 ^o^C. (**b**) U_h_ vs. q_h_ for diff. values of Φ at T = 65 ^o^C. (**c**) U_h_ vs. q_h_ for diff. values of Φ at T = 75 ^o^C.

One of the most important reasons for conducting this research is to know the effect of hybrid nanofluid (HNF) on the heat transfer process in the STHX. Therefore, it is clear in Fig. 5a–c the change in the overall heat transfer coefficient (OHTC) with the flow rate of the HNF with different volume concentrations (Ф) but at a constant temperature for each figure. All figures shown illustrate that the OHTC increases with the increase of HNF flowrate. All experiments in these figures were conducted at a constant flow rate of the cold fluid. The results of the heat transfer process for the HNF with different Ф were compared with those of the conventional fluid (distilled water). The experiments showed that the HNF at all concentrations outperforms the conventional fluid in the heat transfer. The OHTC value increased from 4 to 47.5% according to the Ф value compared with the conventional fluid. The reason for this is that the nanoparticles present in the base fluid, the higher their percentage, the more this contributes to increasing the thermal conductivity of the fluid due to the increase in the Brownian motion of the fluid with the increase in its concentration. It is known that the increase in temperature difference between the two exchange fluids is one of the factors that lead to the improvement of the heat transfer process. The temperature of the fluid was changed in Fig. 5b (65 ^o^C) and c (75 ^o^C) to know the extent of their impact on the increase in temperature and compare them with what happens with water. The results showed that the increase in the temperature of the hot fluid, whether distilled water or the HNFs, increased with the value of the OHTC, but at the same operating factors, it was found that the increase in the temperature of the HNF exceeds the increase resulting from the use of water by values ​​ranging between 5 and 11% depending on the concentration of the fluid and temperature. The reason for this may be due to the increase in the thermal conductivity of the nanofluid with the increase in its temperature by values ​​greater than those of distilled water.

**Fig. 6:**
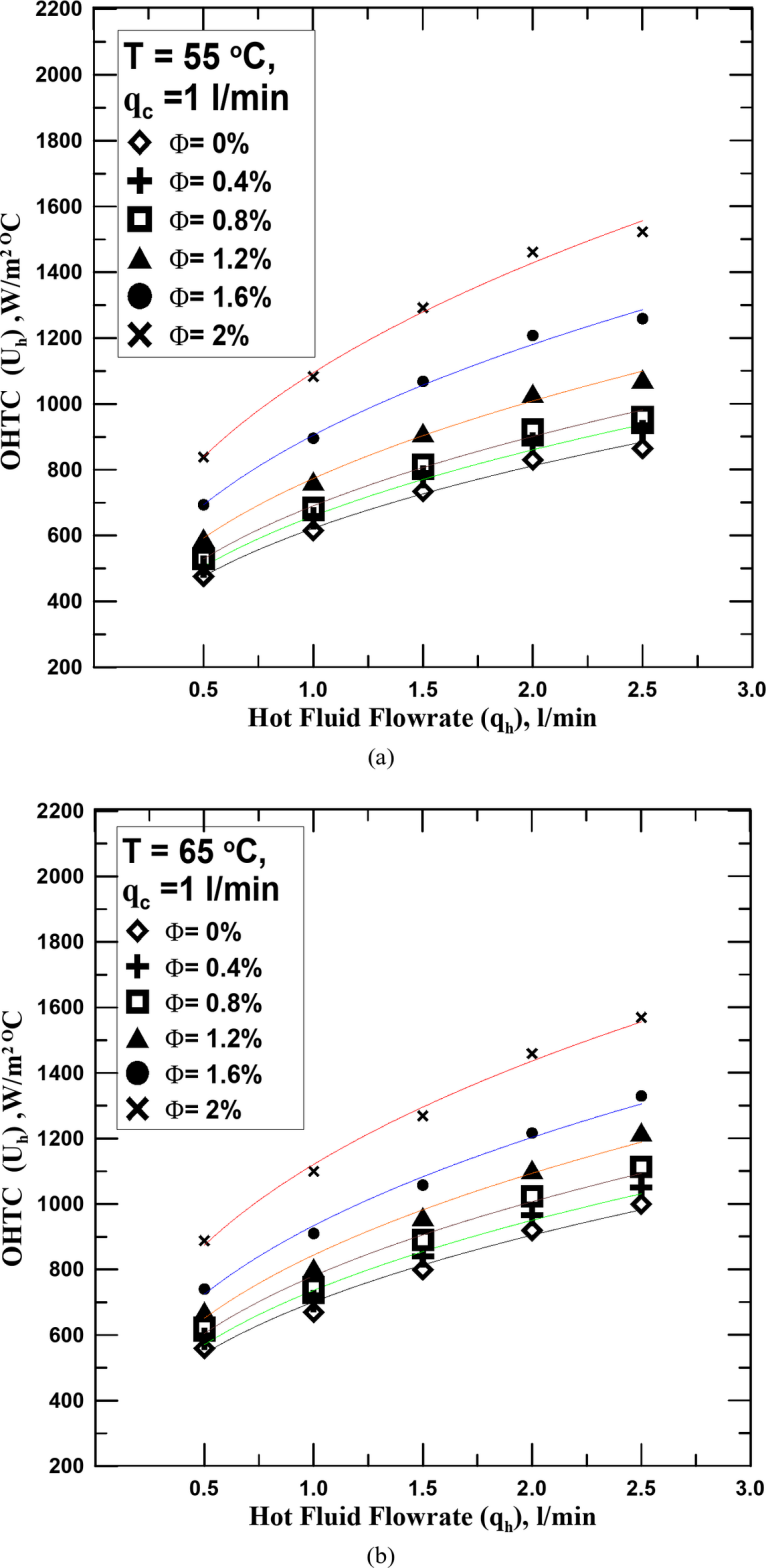

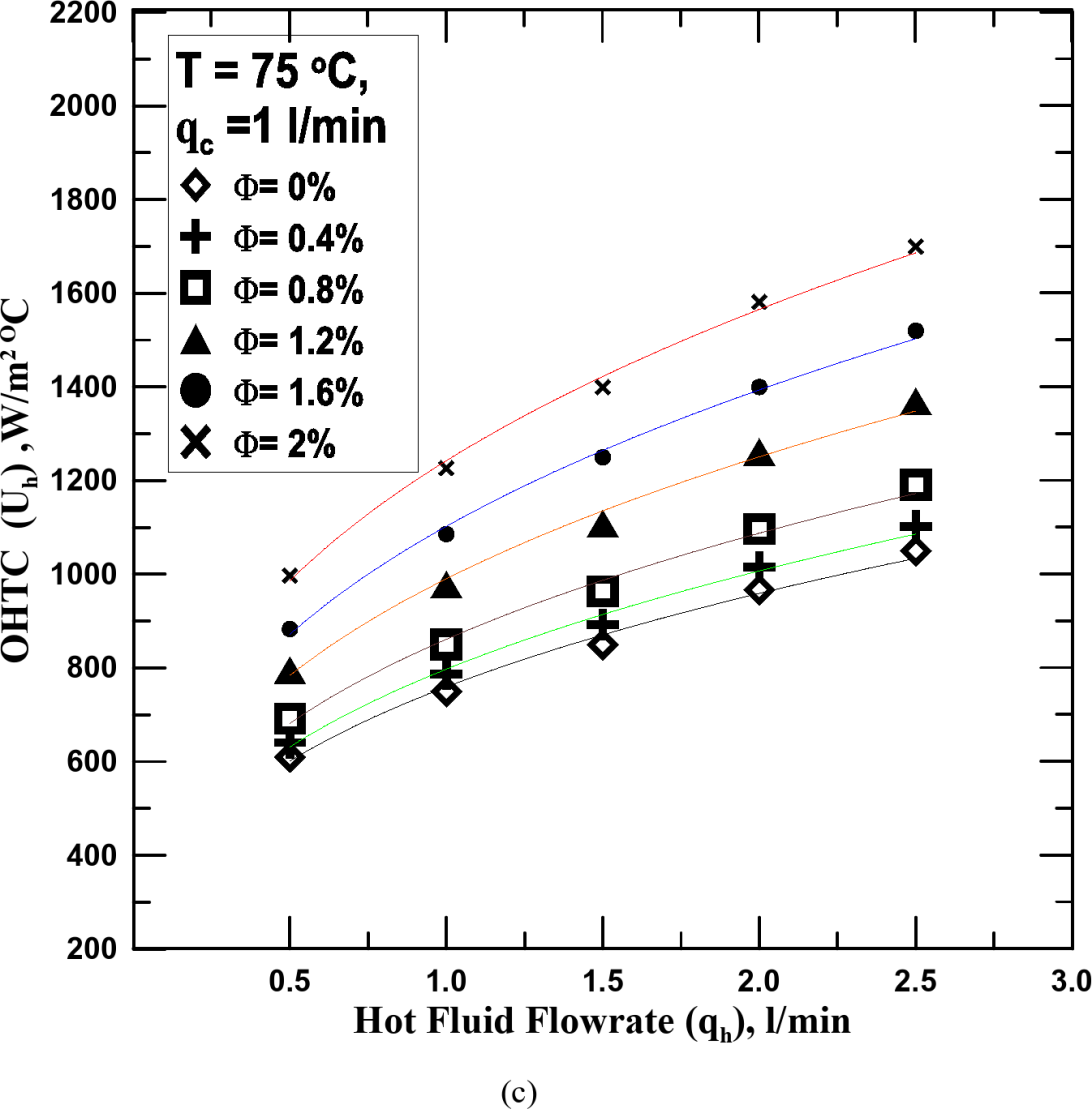
(**a**) U_h_ vs. q_h_ for diff. values of Φ at T = 55 ^o^C. (**b**) U_h_ vs. q_h_ for diff. values of Φ at T = 65 ^o^C. (**c**) U_h_ vs. q_h_ for diff. values of Φ at T = 75 ^o^C.

The variation of the OHTC against the hot fluid flowrate of water and nanofluid with varying Ф is displayed in Fig. 6a–c. The cold fluid flowrate-controlled to be 1 l/min. It is observed that the OHTC increases with the increase of the HNF concentration and increase in cold water flowrate enhances this increase. The OHTC value increased by 11.3–33.72% with a flow rate of 1 l/min compared to that obtained with a flow rate of 0.5 l/min. This is due to the increased heat transfer rate of the cooling water.

**Fig. 7:**
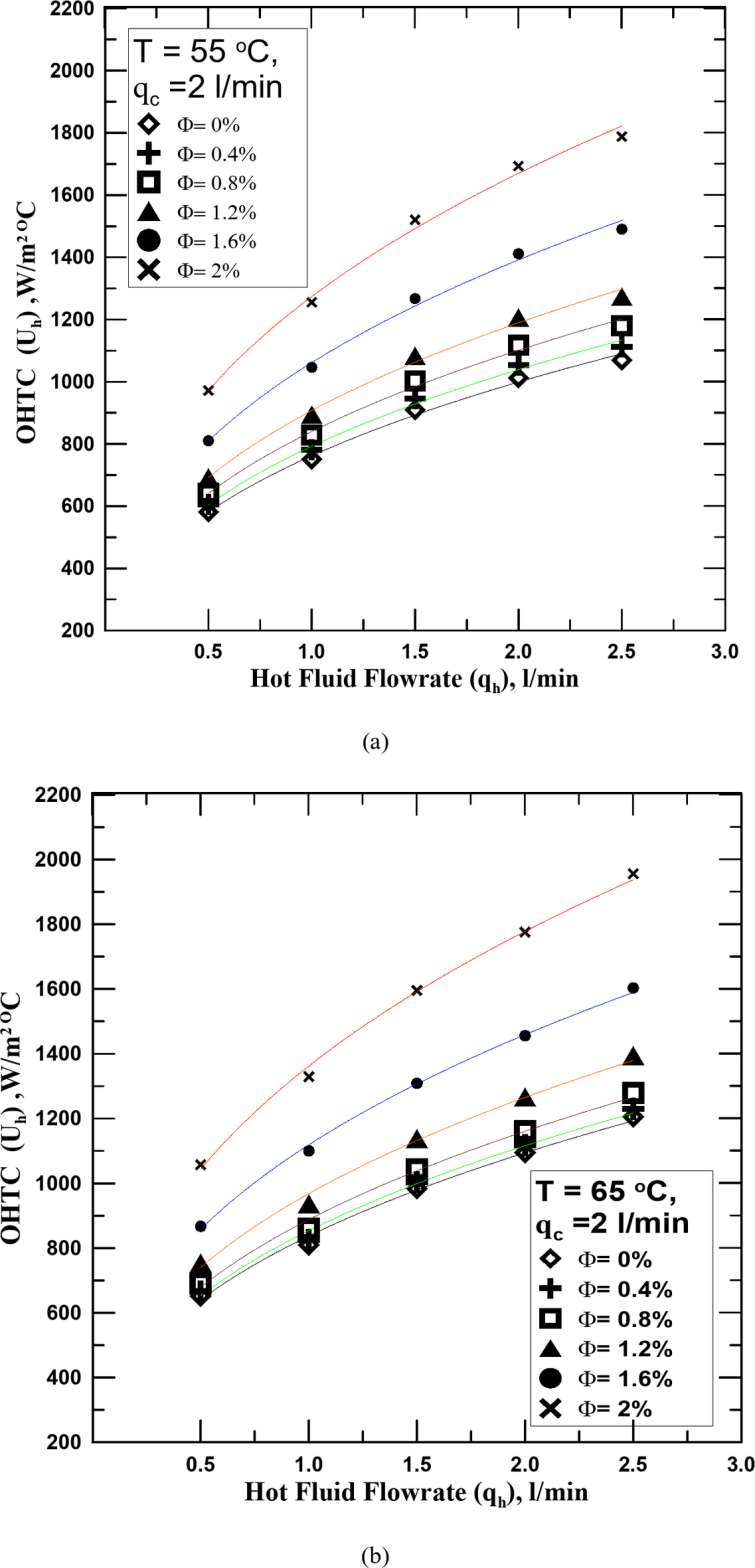

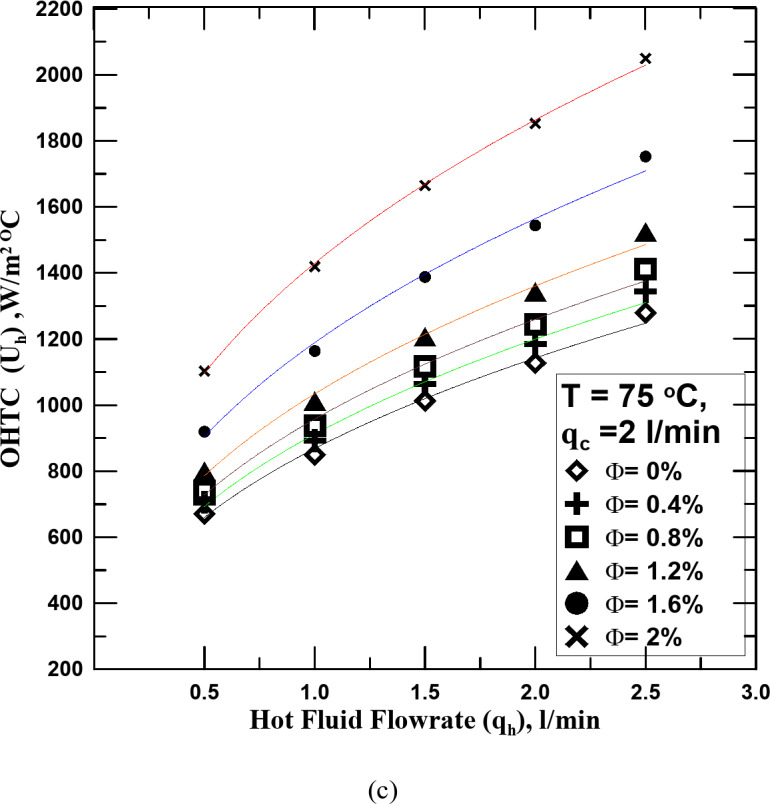
(**a**) U_h_ vs. q_h_ for diff. values of Φ at T = 55 ^o^C. (**b**) U_h_ vs. q_h_ for diff. values of Φ at T = 65 ^o^C. (**c**) U_h_ vs. q_h_ for diff. values of Φ at T = 75 ^o^C.

Figure 7a–c show how the OHTC varies with the hot fluid flowrate of water and nanofluid with different Ф. The regulated cold fluid flowrate was 2 l/min. The same result as in Figs. 5 and 6 is confirmed in this figure with the difference in the rate of increase in the OHTC values ​​with increasing the cold water flowrate.

**Fig. 8:**
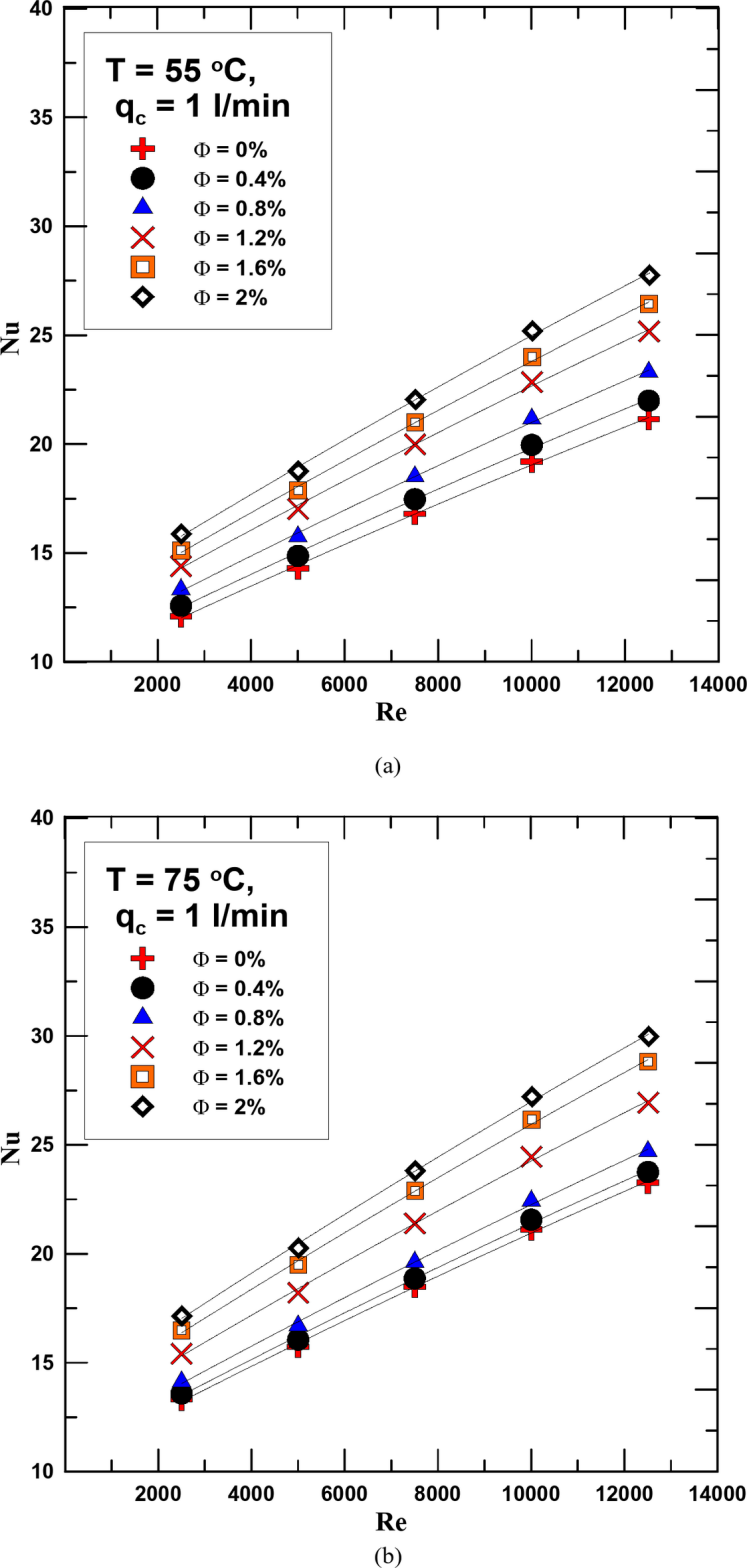
(**a**) Nu versus Re for diff. values of Φ for q_c_ = 2 l/min and T = 55 ^o^C. (**b**) Nu versus Re for diff. values of Φ for q_c_ = 1 l/min and T = 75 ^o^C.

**Fig. 9:**
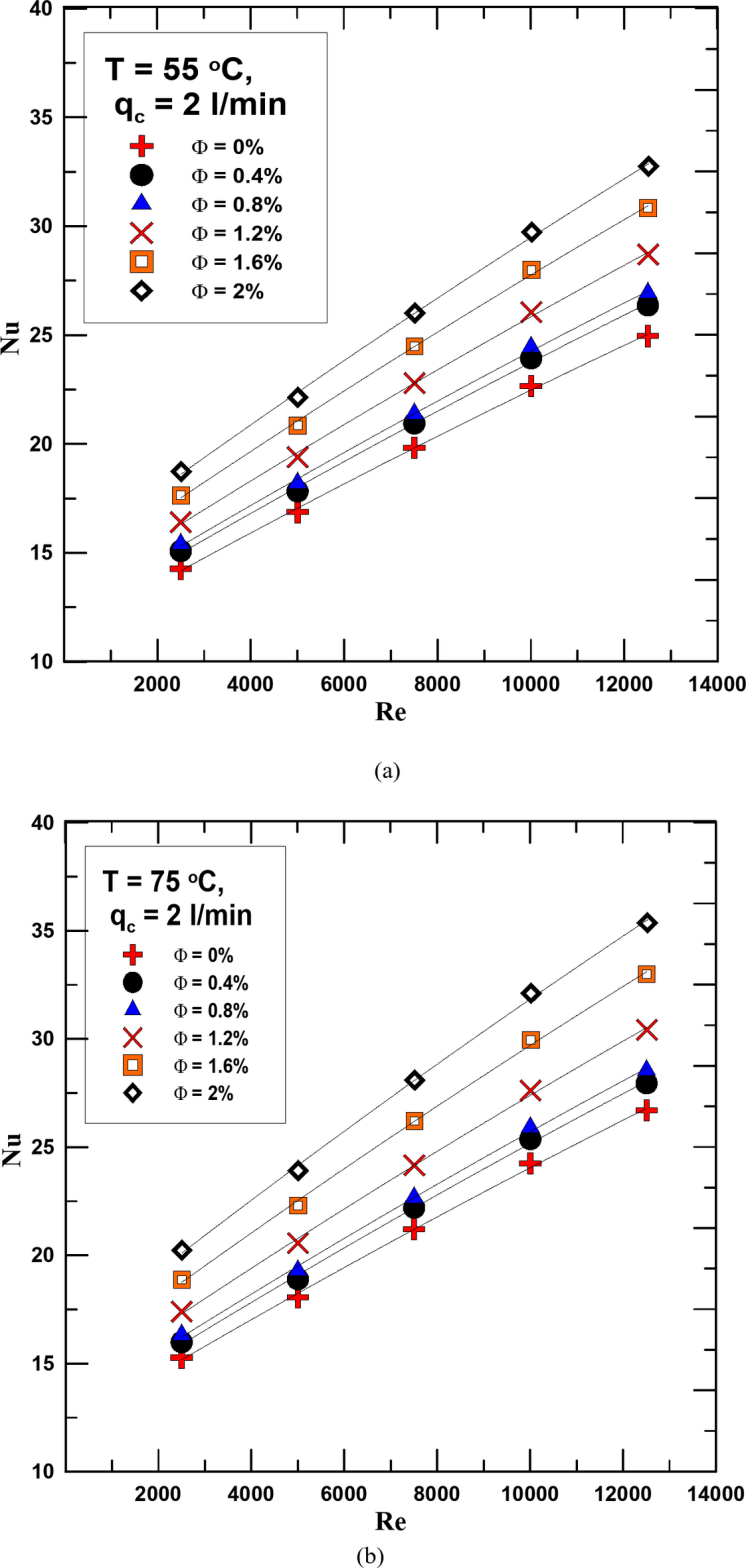
(**a**) Nu versus Re for diff. values of Φ for q_c_ = 2 l/min and T = 55 ^o^C. (**b**) Nu versus Re for diff. values of Φ for q_c_ = 2 l/min and T = 75 ^o^C.

Figures 8 and 9 show the change in Nusselt number (Nu) with the change in Re for different concentrations of the HNF used and distilled water. The relationship was evaluated for the flow rate of cold water 1 and 2 l/min in Figs. 8 and 9 respectively and at hot fluid temperatures of 55 °C and 75 °C. As is clear from the figures shown, the Nu increases with the increase in both the fluid concentration and temperature. The reason for this increase is that due to the increase in the thermal conductivity of the HNF compared to water, this increases the value of the convection heat transfer coefficient, which leads to an increase in the Nusselt number. The Nusselt number increased by 10–28.5% as a result of using the HNF instead of distilled water at a flow rate of 1 l/min for the cold fluid. The percentage increase in value is due to the concentration of the hybrid nanofluid and its temperature. Using a flow rate of 2 l/min also led to an increase in the Nu value of 15% more than using a flow rate of 1 l/min.

**Fig. 10:**
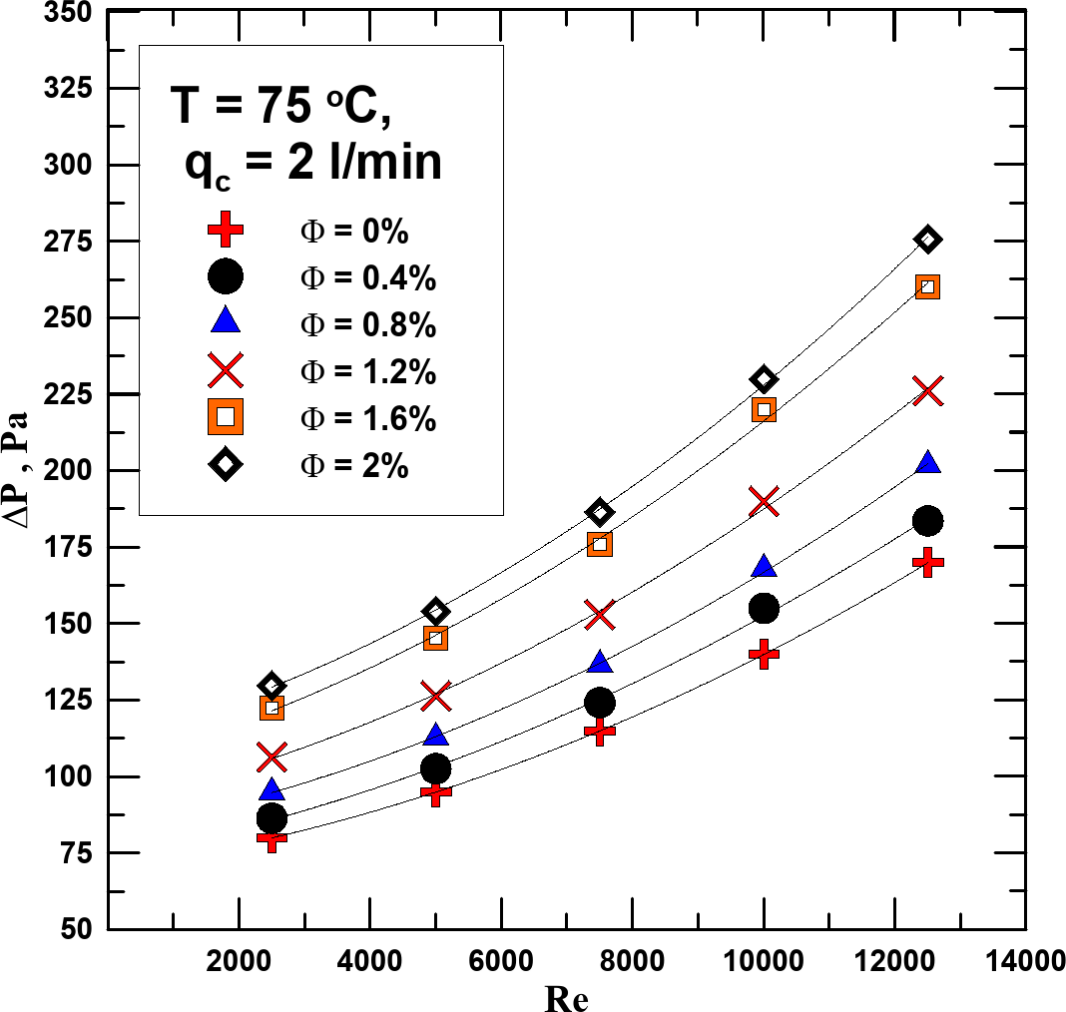
ΔP vs. Re for diff. values of Φ at T = 75 ^o^C.

One of the main factors that must be measured during the experiments is the pressure drop (ΔP) due to flow. Therefore, Fig. 10 shows the change in the ΔP between the hot fluid entering the heat exchanger and its exit with the Reynolds number. The measurement was made for a constant inlet temperature (T = 75 ^o^C.) and a constant cold fluid flow rate (2 l/min). It is clear from the curve shown that the ΔP value increases with the increase in the concentration of the nanofluid (Φ) and the Reynolds number (Re). The reason for this may be that with the increase in the mixing ratio of nanoparticles with the base fluid, the viscosity of the nanofluid increases, which stimulates an increase in the value of the pressure drop. The decrease percentage ranges from 8 to 61.8% depending on the value and Re.

**Fig. 11:**
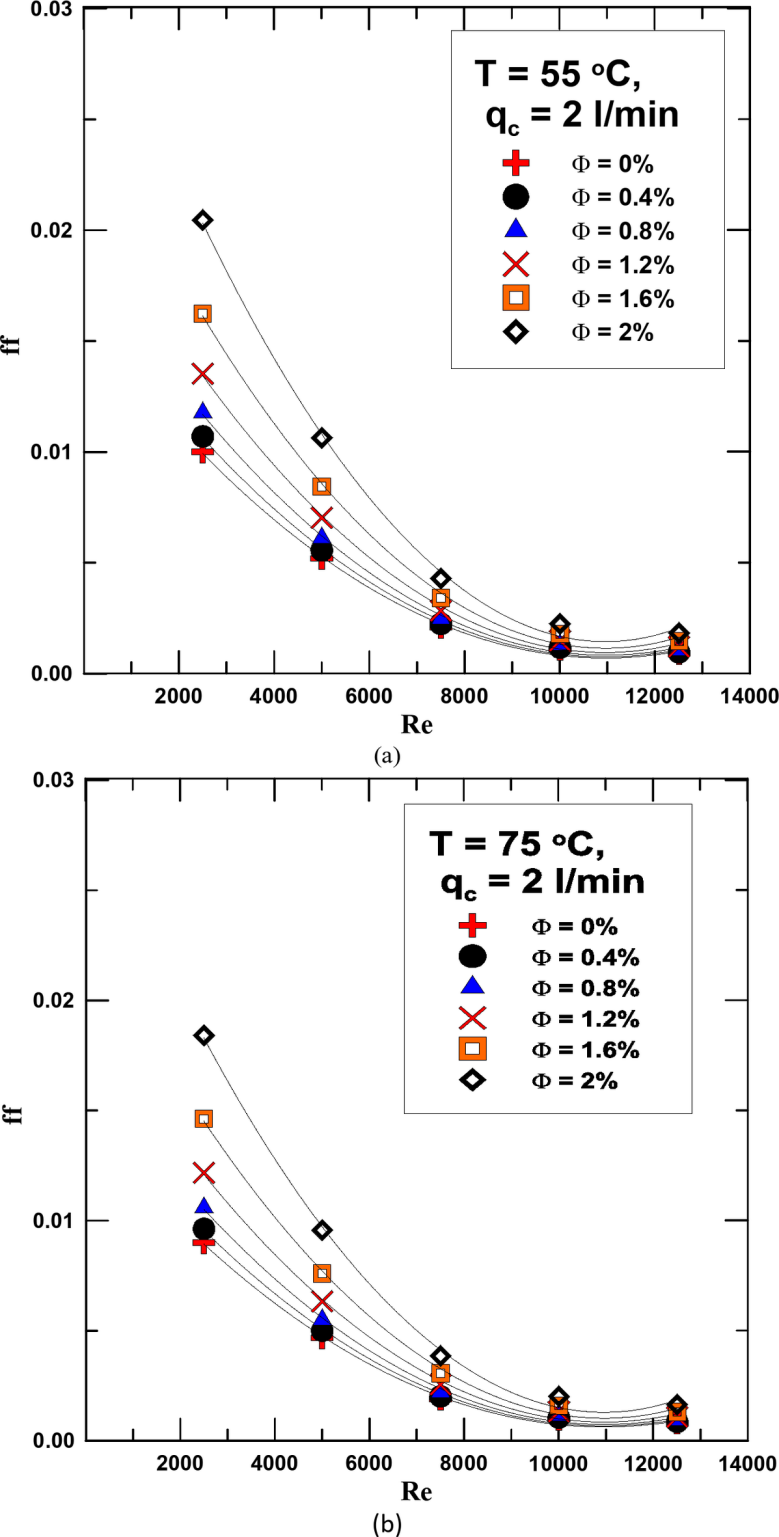
(**a**) ff vs. Re for diff. values of Φ at T = 55 ^o^C. (**b**) ff vs. Re for diff. values of Φ at T = 75 ^o^C.

The friction factor (ff) was calculated in the experiments for all the fluids used and at different temperatures. Figure 11a and b represent the change in the ff with the Re for different concentrations and at temperatures of 55 °C and 75 °C. It is clear from the figure that the ff is greatly affected by the concentration of the fluid used, as its value increases with increasing Φ, due to the increase in the density of the nanofluids compared to distilled water. However, the value of the ff decreases with increasing Re, as the increase in the fluid velocity outweighs the increase in the ΔP. By measuring the value of the increase in the power consumption of the used pump, it was found that there is an increase of up to 12% as a maximum as a result of using hybrid nanofluids. Looking at Fig. 11a and b, we find that the increase in the fluid temperature contributed to the decrease in the ff value due to the decrease in both density and viscosity with increasing temperature. This contributed to a good decrease in the increase in pump power consumption, and also, as mentioned previously, improved the heat transfer process.

**Fig. 12:**
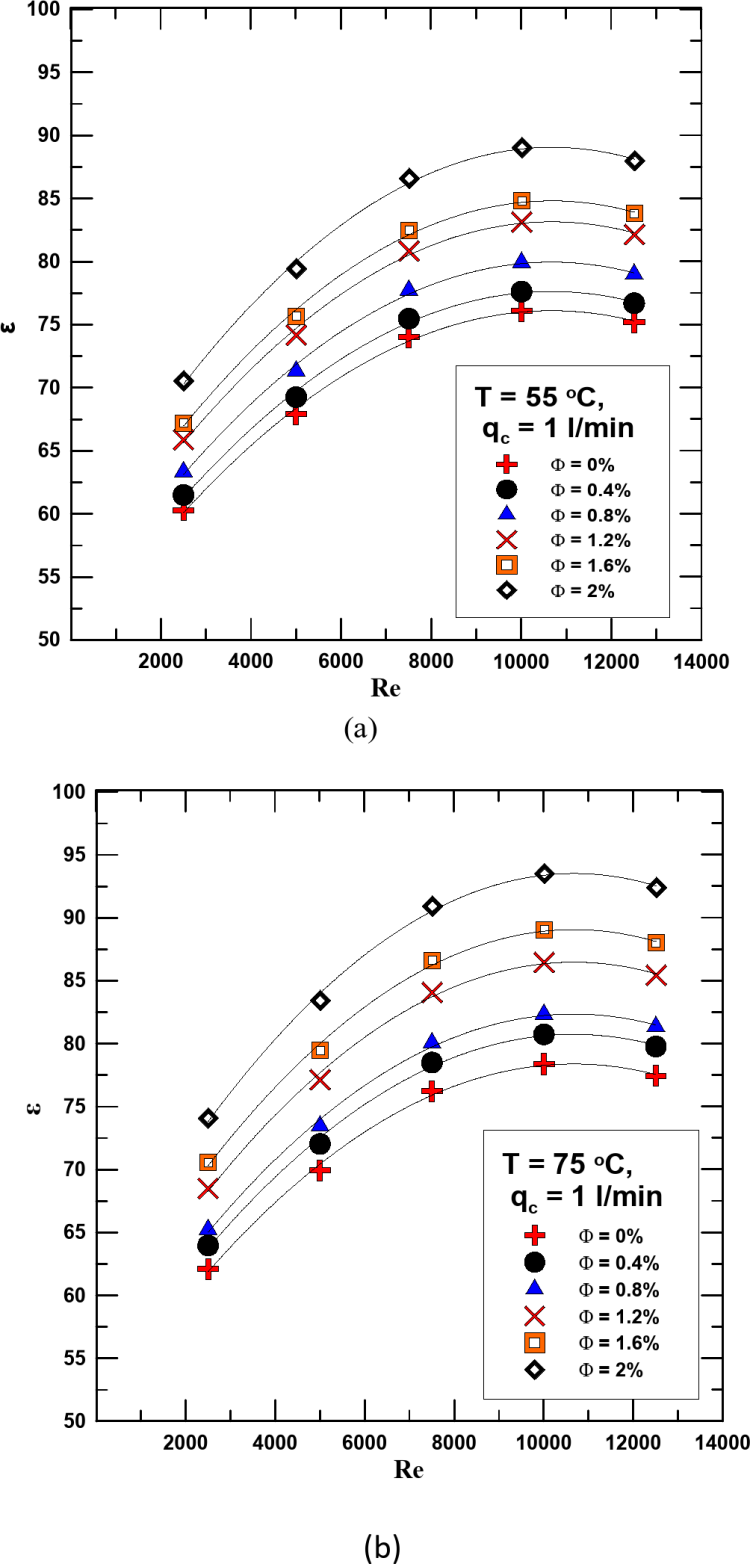
(**a**) ε versus Re for diff. values of Φ for q_c_ = 1 l/min and T = 55 ^o^C. Figure (**b**) ε versus Re for diff. values of Φ for q_c_ = 1 l/min and T = 75 ^o^C.

**Fig. 13:**
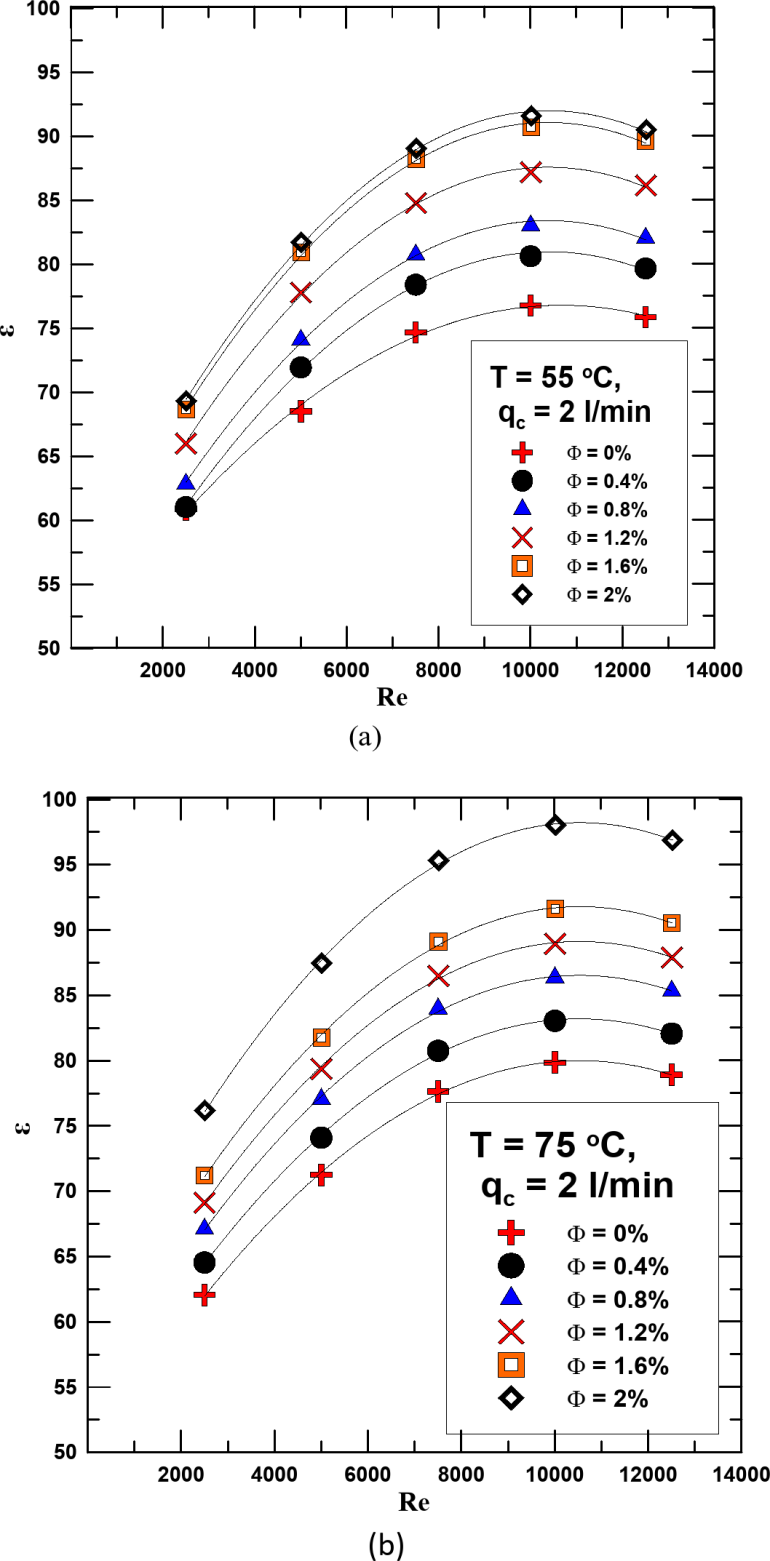
(**a**) ε versus Re for diff. values of Φ for q_c_ = 2 l/min and T = 55 ^o^C. (**b**) ε versus Re for diff. values of Φ for q_c_ = 2 l/min and T = 75 ^o^C.

In Figs. 12 and 13 the effectiveness changes with the Reynolds number for the HNF at different concentrations and for distilled water. The relationship is validated at two different temperatures 55 & 75 °C and at a flow rate of the cold fluid of 1 & 2 l/min. It is clear from the figures that in all cases and at different flow rates, the effectiveness increases with increasing the concentration of the HNF and the Re. Compared to distilled water, it found that the effectiveness increases in the range of 4.1 − 22.7% depending on the concentration and temperature when using HNF.

Through the experiments conducted to test the HNF (AL_2_O_3_ - MWCNT /water) with mixing ratios of 50:50 for all volume concentrations used in the research, and in the Re range of (2500–12560), an experimental correlation was proposed to calculate the Nu value. The proposed correlation is a function of the Re and Φ of the HNF and is valid within the framework of the tested Re and Φ (2500 < Re < 12550 and, 0% < Φ < 2%), for SHTX.

20 $${\text{Nu }} = {\text{ }}2.25{\text{ }}\left( {\text{Re} } \right)^{{0.248}} ~(\Phi )^{{0.129}}$$

**Fig. 14:**
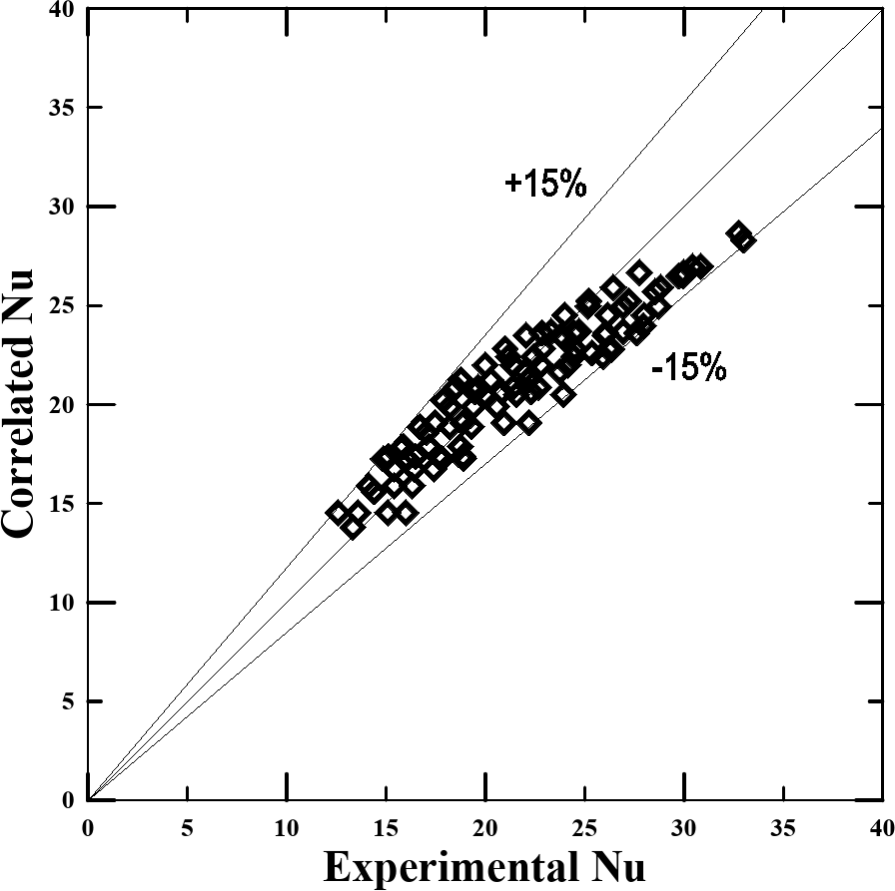
Cal. Nu from the proposed relationship vs. Nu determined from the experimental results.

Figure 14 shows that the expected error rate for using the proposed experimental correlation between the true Nusselt numbers calculated through experiments is within the range of ± 15%.

**Fig. 15:**
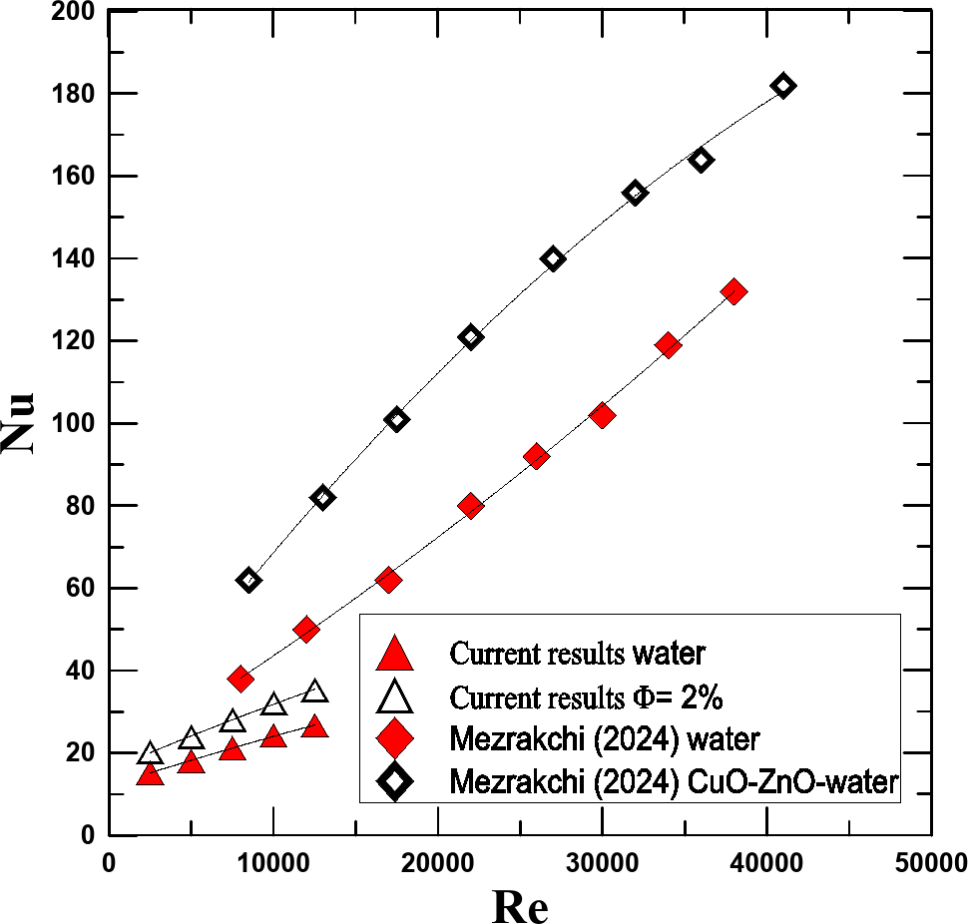
Contrasting Mezrakchi’s [2024] findings with the present Nu versus Re findings.

To verify the validity of the results of the current study, a comparison was made between the results of this work and previous studies. Despite the difference in the hybrid nanofluid used, by referring to the physical properties of both fluids, we find that they are close, so this comparison was made. Within the experimental ranges, by comparing the current research results with what Mezrakchi^31^ reached, it is found an acceptable consistency between both results. Figure 15 shows the change in Nu with Re for both research and by comparison, the reader finds a satisfactory agreement between both results.

## Conclusions

The research on testing nanofluids in different mechanical systems is still under study due to their unique physical properties. The shell and tube heat exchanger is one of the most important types of heat exchangers and the most common due to its advantages. The current study investigated the replacement of the traditional hot fluid, distilled water, with the hybrid nanofluid (MWCNTs- Al_2_O_3_/water) to test its effectiveness and feasibility in this system. A hybrid nanofluid was prepared with different volume concentrations to be a hot working fluid in the heat exchanger. Tests were conducted on these fluids in the range of Re (2500–12560). The effect of changing the temperature of the manufactured fluids was studied and the results were compared with the results issued for the traditional fluid. The most prominent results obtained through the study are the following:

- The hybrid nanofluid has better physical properties than conventional fluids, which makes it more effective in heat transfer. As a result of using this fluid, the OHTC improved by 4-47.5% depending on its concentration compared to distilled water.
- The Nusselt number and effectiveness also increased by using the HNF in different proportions depending on the fluid concentration.
- One of the negative points in using the HNF is the increased pressure drop and friction factor due to its increased density and viscosity compared to conventional fluids.
- The pump’s energy consumption capacity also increases, and the percentage of increase also varies according to the fluid concentration.
- The following experimental correlation was concluded from the results obtained from this current study to calculate the Nu as a function of Re and Φ of the HNF (MWCNTs- Al_2_O_3_/water) at mixing ratios of (50:50) and within the mentioned Re range.

Nu = 2.25 (Re)^0.248^ (Φ)^0.129^.

## Data Availability

The datasets used and/or analyzed during the current study are available from the corresponding author on reasonable request.

## List of symbols

A: Surface area, m^2^
C: Specific heat, J/kg. K
D: Diameter, m
ff: Fraction factor, -
h: Convection heat transfer coefficient, W/ m^2^
k: Thermal conductivity, W/m. K
U: Overall heat transfer coefficient, W/ m^2^ .K
V: Velocity, m/s
m: Mass flow rate, kg/s
Q: Rate of heat dissipated, W
q: Volume flow rate, l/min
Re: Reynolds number,$\:\:(-)$
Nu: Nusselt number, ( $\:-)$
T: Temperature, K

## Greek symbols

µ: Absolute viscosity, kg/(m·s)
Φ: Volume concentration,-
ρ: Density kg/m^3^

## Abbreviations

HEs: Heat exchangers
HNF: Hybrid nanofluids
PHE: Plate heat exchanger
RHT: Rate of heat transfer
OHTC: Overall heat transfer coefficient
STHX: Shell and tube heat exchanger
TEM: Transmission electron microscopy

## Subscripts

b: Base
c: Cold
f: Fluid
h: Hot, hydraulic
i: Inner, inlet
o: Outer, outlet
p: Particles
t: Total
w: Water
